# Unit policies regarding tocolysis after preterm premature rupture of membranes: association with latency, neonatal and 2-year outcomes (EPICE cohort)

**DOI:** 10.1038/s41598-020-65201-y

**Published:** 2020-06-12

**Authors:** Elsa Lorthe, Carla Moreira, Tom Weber, Lene D. Huusom, Stephan Schmidt, Rolf F. Maier, Pierre-Henri Jarreau, Marina Cuttini, Elizabeth S. Draper, Jennifer Zeitlin, Henrique Barros, The EPICE research group, E. Martens, G. Martens, P. Van Reempts, K. Boerch, A. Hasselager, L. Huusom, O. Pryds, T. Weber, L. Toome, H. Varendi, P.-Y. Ancel, B. Blondel, A. Burguet, P.-H. Jarreau, P. Truffert, R. F. Maier, B. Misselwitz, S. Schmidt, L. Gortner, D. Baronciani, G. Gargano, R. Agostino, I. Croci, F. Franco, V. Carnielli, M. Cuttini, D. DiLallo, C. Koopman-Esseboom, A. Van Heijst, J. Nijman, J. Gadzinowski, J. Mazela, L.-M. Graça, M.-C. Machado, C. Rodrigues, T. Rodrigues, H. Barros, A-K. Bonamy, M. Norman, E. Wilson, E. Boyle, E. S. Draper, B. N. Manktelow, A. C. Fenton, D. W. A. Milligan, J. Zeitlin, M. Bonet, A. Piedvache

**Affiliations:** 1 EPIUnit – Institute of Public Health, University of Porto, Porto, Portugal; 2 Department of Mathematics and Applications, University of Minho, Campus de Gualtar, 4710-057 Braga, Portugal; 3 University of Copenhagen, Copenhagen, Denmark; 4 Department of Obstetrics and Gynaecology, Hvidovre University Hospital, Kettegård Allé 30, 2650 Hvidovre, Denmark; 5 Department of Obstetrics, University of Marburg, Marburg, Germany; 6 Children’s Hospital, University Hospital, Philipps University Marburg, Marburg, Germany; 7 Université de Paris, Epidemiology and Statistics Research Center/CRESS, INSERM, INRA, F-75004 Paris, France; 8 Service de Médecine et Réanimation Néonatales de Port-Royal, Hôpitaux Universitaires Paris Centre, AP-HP, Paris, France; 9 Clinical Care and Management Innovation Research Area, Bambino Gesù Children’s Hospital, IRCCS, Rome, Italy; 10 Department of Health Sciences, University of Leicester, Leicester, United Kingdom; 11 Departamento de Ciências da Saúde Pública e Forenses e Educação Médica, Faculdade de Medicina, Universidade do Porto, Porto, Portugal; 12 Study Centre for Perinatal Epidemiology, Brussels, Belgium; 13 Department of Neonatology, Antwerp University Hospital, University of Antwerp, Antwerp, Belgium; 14 Department of Paediatrics, Hvidovre Hospital, Copenhagen University Hospital, Hvidovre, Denmark; 15 Department of Neonatology, Hvidovre University Hospital, Hvidovre, Denmark; 16 Tallinn Children’s Hospital, Tallinn, Estonia; 17 University of Tartu, Tartu, Estonia; 18 Division of Pediatrics 2, Hôpital du Bocage; INSERM CIE1, CHRU Dijon, Université de Dijon, Dijon, France; 19 Department of Neonatology, Jeanne de Flandre Hospital, Lille CHRU, Lille, France; 20 Institute of Quality Assurance, Hesse, Germany; 21 Department of Neonatology, Pediatric Center, Justus Liebig University Giessen, Giessen, Germany; 22 Hospital Care Services, General Directorate for Health and Social Policies, Emilia Romagna Region, Bologna, Italy; 23 Neonatal Intensive Care Unit, Department of Obstetrics and Paediatrics, S. Maria Nuova Hospital, IRCCS, Reggio Emilia, Italy; 24 Department for Mother’s and Infant’s Health, Hospital San Giovanni Calibita - Fatebenefratelli, Rome, Italy; 25 Health Department, Regione Lazio, Italy; 26 Maternal and Child Health Institute, Marche University and Salesi Hospital, Ancona, Italy; 27 Department of Neonatology, Wilhelmina Children’s Hospital, Utrecht, The Netherlands; 28 Department of Neonatology, Radboud University Medical Center, Nijmegen, The Netherlands; 29 Department of Neonatology, Poznan University of Medical Sciences, Poznan, Poland; 30 Department of Neonatology and Neonatal Infectious Diseases, Poznan University of Medical Sciences, Poznan, Poland; 31 Department of Obstetrics and Gynecology, Santa Maria University Hospital, Faculty of Medicine, University of Lisbon, Lisbon, Portugal; 32 Lisbon Academic Medical Center, University of Lisbon, Lisbon, Portugal; 33 Department of Gynecology and Obstetrics, Centro Hospitalar S. João, Porto, Portugal; 34 Clinical Epidemiology Unit, Department of Medicine Solna; Department of Women’s and Children’s Health, Karolinska Institutet, Stockholm, Sweden; 35 Department of Clinical Science, Intervention and Technology, Division of Pediatrics, Karolinska Institutet; and Department of Neonatal Medicine, Karolinska University Hospital, Stockholm, Sweden; 36 Department of Clinical Science, Intervention and Technology, Karolinska Institutet, Stockholm, Sweden; 37 Newcastle University, Newcastle, United Kingdom

**Keywords:** Health policy, Epidemiology, Paediatric research

## Abstract

After preterm premature rupture of membranes (PPROM), antibiotics and antenatal steroids are effective evidence-based interventions, but the use of tocolysis is controversial. We investigated whether a unit policy of tocolysis use after PPROM is associated with prolonged gestation and improved outcomes for very preterm infants in units that systematically use these other evidence-based treatments. From the prospective, observational, population-based EPICE cohort study (all very preterm births in 19 regions from 11 European countries, 2011–2012), we included 607 women with a singleton pregnancy and PPROM at 24–29 weeks’ gestation, of whom 101, 195 and 311 were respectively managed in 17, 32 and 45 units with no-use, restricted and liberal tocolysis policies for PPROM. The association between unit policies and outcomes (early-onset sepsis, survival at discharge, survival at discharge without severe morbidity and survival at two years without gross motor impairment) was investigated using three-level random-intercept logistic regression models, showing no differences in neonatal or two-year outcomes by unit policy. Moreover, there was no association between unit policies and prolongation of gestation in a multilevel survival analysis. Compared to a unit policy of no-use of tocolysis after PPROM, a liberal or restricted policy is not associated with improved obstetric, neonatal or two-year outcomes.

## Introduction

Preterm premature rupture of membranes (PPROM) is responsible for one third of all preterm births and is associated with a high rate of perinatal mortality and morbidity, related to prematurity and specific complications such as intra-uterine infection^1–4^. In this clinical setting, evidence-based interventions include the use of antibiotics, shown to prolong pregnancy and improve short-term neonatal morbidity^5–8^, and antenatal steroids, associated with reductions in short-term neonatal mortality and morbidity^9^. Moreover, for PPROM occurring before 34 weeks’ gestation in the absence of labor, chorioamnionitis or fetal distress, expectant management is usually recommended to reduce prematurity and its adverse neonatal consequences^10–14^.

Tocolytic treatments are widely administered after very preterm PROM^15–18^. They aim at prolonging gestation to allow for gains in fetal maturity as well as administration of antenatal steroids and in utero transfer. However, they may also prolong fetal exposure to deleterious inflammation and/or infection and obstetric complications (such as cord prolapse or placental abruption). Evidence of their benefit is lacking, with no demonstrated improvement in neonatal outcomes and controversial results regarding the prolongation of gestation^18,19^. While tocolysis was associated with longer gestation in some randomized controlled trials performed more than 25 years ago^20–23^, this was not found in the most recent study^18^. Changes in antenatal management, namely the widespread use of antibiotics, could have modified the association between tocolysis and prolongation of pregnancy after PPROM.

This study used data from the European population-based project on ‘Effective Perinatal Intensive Care in Europe’ (EPICE) to assess whether unit policy regarding tocolysis after PPROM was associated with prolonged latency until delivery and improved neonatal and two-year outcomes of very preterm infants born in maternity units with policies of systematic use of both antibiotics and antenatal corticosteroids for PPROM.

## Materials and Methods

### Study design

This is a secondary analysis of the EPICE cohort, a prospective, population-based study implemented to assess the use of evidence-based medicine for the care of very preterm infants^24^. Eligible participants included all live births, stillbirths and terminations of pregnancy at 22^0/7^ to 31^6/7^ weeks’ gestation that occurred in all public and private maternity units in 19 regions from 11 European countries, covering around 850,000 annual births: Belgium (Flanders), Denmark (Eastern region), Estonia (entire country), France (Burgundy, Ile de France, North-Pas-de-Calais), Germany (Hesse, Saarland), Italy (Emilia-Romagna, Lazio, Marche), the Netherlands (Eastern-Central region), Poland (Wielkopolska), Portugal (Lisbon, Northern region), Sweden (greater Stockholm area) and the United Kingdom (East Midlands, Northern, Yorkshire and Humber regions). Recruitment started between March and July 2011 and the inclusion period lasted 12 months, except in France where it lasted 6 months. Initial follow-up was performed until discharge home from hospital or into long-term care or death. Investigators abstracted maternal, obstetric and neonatal data from medical records using common definitions and a pretested standardized questionnaire. Follow-up at 2 years of corrected age aimed at assessing longer term health outcomes, in particular growth, motor and cognitive development, and was based on parental questionnaires. This questionnaire was developed in English, translated into national languages, back-translated and pretested by the country teams. Data on policies and usual practices related to medical interventions and decision making were contemporaneously collected in the spring of 2012, as part of the EPICE study, by use of a structured questionnaire sent to heads of all maternity units associated with neonatal units with at least 10 very preterm admissions during the study period^24^.

### Patient involvement

The project consortium includes a European parent organization, and maintains contact with families participating in the cohort through its website and regional coordination teams (birthday letters, newsletters).

### Study population

The present study included all singleton pregnancies diagnosed with PPROM at 24^0/7^–29^6/7^ weeks’ gestation, defined as spontaneous rupture of membranes at least 12 hours before delivery, and delivered at 24^0/7^–31^6/7^ weeks. Women with PPROM at 30–31 weeks were excluded from this analysis because according to the cohort’s design, only those delivering before 31^6/7^ weeks were eligible to participate in the EPICE cohort. Exclusion criteria were serious congenital anomalies, as reported by the European Registry of Congenital Anomalies (EUROCAT) and detailed previously^25^, and in utero fetal demise before the diagnosis of PPROM. We also excluded all cases born in a maternity unit where antibiotics and antenatal steroids were not systematically prescribed in this clinical setting. Finally, infants born in units with no policy or missing data regarding the use of tocolysis after PPROM were excluded from this analysis.

### Variables

#### Unit variables

Unit variables were reported by each maternity unit in a specific questionnaire and each patient was assigned the same values as other patients in the same institution. The main exposure was the declared unit policy regarding the use of tocolysis after PPROM, reported as liberal (‘whenever possible’), restricted (‘sometimes’) or no-use (‘no’). Among units with a liberal use of tocolysis, we further investigated policies for the first line tocolytic (betamimetic [BM], oxytocin antagonist [OA] vs calcium channel blocker [CCB]) and the length of use (as long as necessary vs ≤48 hours). One single maternity unit (with 9 participants in this study) used magnesium sulfate as first line tocolytic, and was excluded from this specific analysis.

Maternity unit-level variables consisted of unit characteristics (level of care [level III, with an onsite NICU, vs levels I-II], status [public vs non-public], unit size [defined by the number of births in 2011], overall cesarean section rate in 2011 [by quartile], participation in a perinatal network), and responses to questions about protocol development and assessment in the unit (existence of written unit protocols, implementation of audits or routine data collection on compliance to protocols) and scientific activity (participation in clinical trials, meetings to discuss scientific publications).

#### Patient-level variables

We assessed the association of unit policy with perinatal outcomes, namely early-onset sepsis (defined as positive bacterial culture in blood or cerebrospinal fluid, associated with clinical and/or laboratory signs of infection, during the first three days of life), survival at discharge and survival at discharge without severe morbidity. Severe morbidity was a composite score including severe forms of intraventricular hemorrhage (IVH grades III or IV), and/or cystic periventricular leukomalacia (cPVL), and/or surgical necrotizing enterocolitis (NEC requiring surgical treatment or peritoneal drainage) and/or retinopathy of prematurity (ROP stage 3 or greater). Severe bronchopulmonary dysplasia (BPD) was the need for 30% or more oxygen and/or respiratory support at 36 weeks of postmenstrual age. However, we chose not to include BPD in our composite score because data on the fraction of inspired oxygen (FiO_2_) was not collected in two regions in the UK. Another outcome was latency duration. This was defined by the time between rupture of membranes and delivery in days. Finally, we investigated survival at two years of corrected age without gross motor impairment as a secondary outcome. Parents were asked five forced-choice items from which impairment in gross motor function, hearing and vision were classified using standard criteria from a scale which has been used in clinical practice across the UK since the late 1990s^26^. Gross motor impairment was defined as inability to walk without assistance or aids, inability to sit without support or inability to hold the head up^27^. Severe hearing impairment was classified if the child was deaf or had functional hearing loss requiring correction with aids but still had difficulty hearing, and severe visual impairment if the child was blind or able to see light only.

Gestational age (GA) was defined as the best estimate of the obstetrical team, based on the last menstrual period and the first-trimester ultrasound assessment, which is part of routine obstetric care in all regions^24^. Small for GA (SGA) was defined as birth weight ≤10th percentile according to intrauterine growth curves, based on weight in each country collected in the Euro-Peristat project and modeled using Gardosi’s formula^28^.

Although the core outcome set for the prevention of preterm birth was not established yet when the study was designed, all the components of the neonatal set of outcomes were collected and used to define the outcomes of the present analysis^29^.

### Statistical analysis

We first described the policies regarding the use of tocolysis after PPROM by country. Demographic, obstetric and unit characteristics were reported as percentages with 95% confidence intervals (95% CI) or medians with interquartile range (IQR) and were compared by unit policy, using chi-square or Fisher’s exact tests as appropriate for categorical variables and nonparametric equality-of-medians tests for quantitative variables.

The association between unit policies and latency duration (considered as a continuous variable) was investigated using survival analysis. Follow-up time was calculated from PPROM to delivery, i.e. equivalent to the latency duration. There were no censored data as all women were included based on gestational age at birth. Survival curves of latency duration by unit’s policy were plotted using the Kaplan-Meier method and compared with a log rank test. We then used three-level survival analysis to account for within-cluster (defined as maternity units and countries) homogeneity in outcomes, with a log-normal distribution of the hazard function^30^. As assumptions of proportionality, tested using the Schoenfeld residual test, were violated, we used a stratified Cox model. Stratification offers a way of extending the Cox proportional hazard model to allow for covariates with non-proportional hazards, and for different baseline hazards for each level of the variable while providing estimates of hazard ratios that are the same for each stratum. Results were reported as hazard ratios (HR) with 95% CI. Known risk factors for shortened latency duration among singleton pregnancies include uterine contractions, cervical changes, oligohydramnios and the occurrence of any complication including infection^18^. We had no exhaustive individual data about the two former and the two latter were considered as intermediate factors. Final models were thus adjusted for gestational age at PPROM, which is a relevant potential confounder issued from the literature.

The association between unit policy regarding the use of tocolysis after PPROM and neonatal and 2-year outcomes was investigated using three-level random-intercept logistic regression models, to take into consideration the hierarchical structure of our data (correlation between individual observations within maternity units considered as level two, themselves nested within countries considered as level three). We hypothesized that the probability of the outcome randomly varies across clusters (random intercept), and that the effect of unit’s policy is equal across clusters (fixed effect)^30^. Multivariate models were adjusted for unit characteristics (hospital size) and individual characteristics (gestational age at PPROM), which are relevant potential confounders issued from the literature^31^. The variable ‘antenatal steroids’ was not included in multivariate models as it can be considered an intermediate variable between the policy regarding the use of tocolysis after PPROM and neonatal outcomes. Results were reported as odds ratios (OR) with 95% CI. We reported intra-class correlations (ICC) that measure the observational cluster effect. Expressed as a percentage, the value of the ICC goes from 0% to 100%, and is close to 0% if the units or the countries are not relevant for understanding individual outcomes differences.

The proportion of missing data was lower than 3% for all covariates, except for the mother’s country of birth (12%) and magnesium sulfate (7%). However, attrition was substantial in the EPICE cohort study: of the 545 children alive at 2 years’ corrected age, 198 (36.3%) were lost to follow-up. The proportion of infants lost to follow-up was different by country, ranging from 0% in Estonia to 65% in the UK (p < 0.001). Other characteristics associated with loss to follow-up, after taking into account the country and the unit, were non liberal policy for tocolysis, younger and foreigner mothers and multiparity (Table S1). We performed multiple imputations with chained equations (with logistic regression, ordered logit regression models and predictive mean matching for missing binary, ordinal categorical and continuous data, respectively) using variables potentially predicting loss to follow-up and/or outcomes^32,33^. These variables were maternal characteristics (age, country of birth, parity), obstetric characteristics (gestational age at PPROM and at birth, antenatal steroids, mode of delivery), neonatal characteristics (SGA, sex, all outcomes defined in this study) and unit characteristics (level, size and policy regarding tocolysis). Associations were estimated within each of the 50 imputed data sets generated with 20 iterations, and results were pooled according to Rubin rules. Statistical significance was set at two-tailed p < 0.05. Analyses were carried out using Stata/SE 13.0 (StataCorp LP, College Station, TX, USA) and R-3.5.0 (coxme package).

### Ethics approval

Parental informed consents were obtained in accordance with national legislations. In addition to ethics approvals from regional or hospital ethics committees, ethics authorization for the European study was obtained from the French Advisory Committee on Use of Health Data in Medical Research (CCTIRS N° 13.020 on 24/01/2013) and the French National Commission for Data Protection and Liberties (CNIL DR-2013-194, on 10/04/2013).

## Results

**Figure 1:**
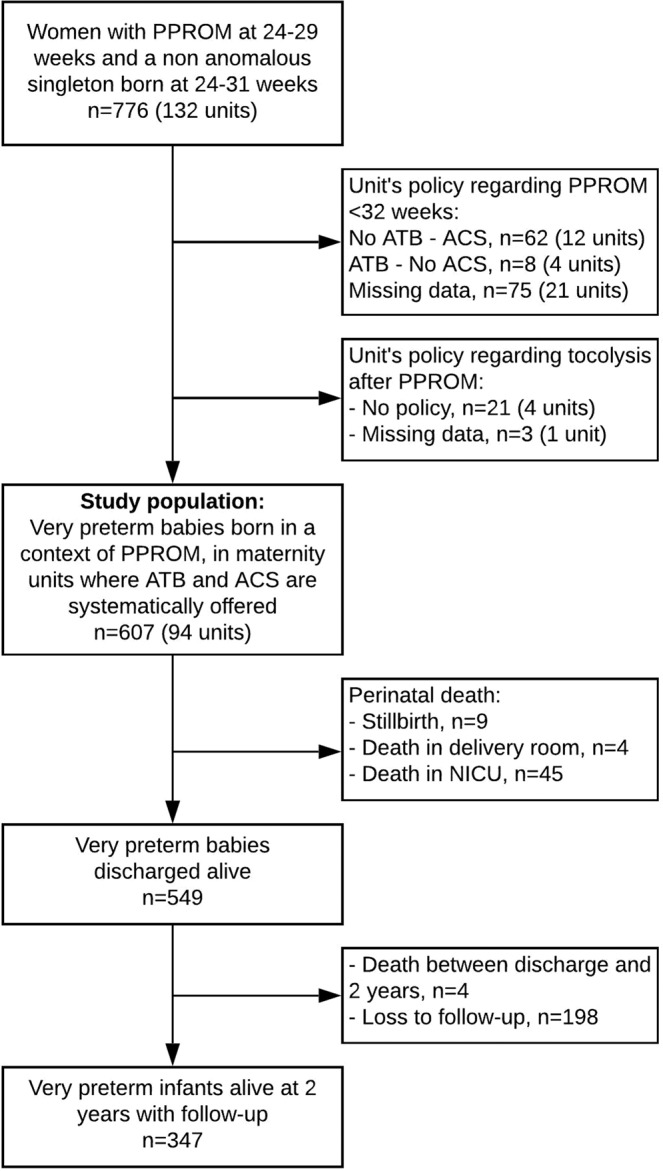
Flow Chart. ATB: antibiotics, ACS: antenatal corticosteroids, NICU: neonatal intensive care unit, PPROM: preterm premature rupture of membranes. Some units had different policies for ACS administration according to gestational age at PPROM, the policy of ACS was then defined at an individual level according to both unit policy and GA at PPROM, explaining slight differences in the total number of units excluded and the number of units after exclusions.

We included 607 women with PPROM at 24^0/7^-29^6/7^ weeks’ gestation who gave birth in 94 maternity units where antibiotics and antenatal steroids were systematically offered in this clinical setting (Fig. 1). Among them, 101, 195 and 311 were respectively managed in 17, 32 and 45 units with no-use, restricted and liberal policies regarding tocolysis after PPROM.

### Unit policies

There was a large variability in policies between and within countries (Supplementary Fig. S1). In France, Italy, Portugal and the United Kingdom, the three policies were represented across maternity units, whereas most units had a liberal policy in the Netherlands, Estonia, Poland, Belgium and Germany, and a restricted policy in Denmark and Sweden. Among units with a liberal policy, 46.5%, 30.2% and 23.3% prescribed OA, CCB and BM as first line tocolytic treatments, respectively, and 71.1% used tocolysis for a maximum of 48 hours after PPROM.

Most unit-level characteristics were not associated with tocolysis unit policy, in particular the level and status of the unit (Table 1). However, units participating in clinical trials or with routine data collection on compliance to protocols were more likely not to use tocolysis after PPROM. Units with meetings to discuss scientific publications more often had a policy of no-use or liberal use of tocolysis after PPROM.

**Table 1:** Association of unit characteristics and unit policy of tocolysis after PPROM.

	Unit policy regarding the use of tocolysis after PPROM			p-value
	No-use (n = 17 units)	Restricted (n = 32 units)	Liberal (n = 45 units)	
**Unit characteristics**				
Number of births in 2011 (n = 94)				
≤1999	2 (11.7)	6 (18.8)	12 (26.7)	0.13
2000–2999	2 (11.8)	6 (18.7)	15 (33.3)	
3000–3999	6 (35.3)	8 (25.0)	11 (24.4)	
4000–4999	2 (11.8)	8 (25.0)	4 (8.9)	
≥5000	5 (29.4)	4 (12.5)	3 (6.7)	
Overall cesarean section rate in 2011 (n = 92)				
1^st^ quartile (15.50%-21.09%)	4 (23.5)	9 (28.1)	10 (23.2)	0.44
2^nd^ quartile (21.10%-23.91%)	6 (35.3)	10 (31.3)	7 (16.3)	
3^rd^ quartile (23.92%-31.90%)	4 (23.5)	8 (25.0)	11 (25.6)	
4^th^ quartile (31.91%-58.50%)	3 (17.7)	5 (15.6)	15 (34.9)	
Level III maternity unit (n = 94)	11 (64.7)	24 (75.0)	36 (80.0)	0.46
Public status (n = 92)	17 (100.0)	30 (93.8)	39 (90.7)	0.65
Part of a network (n = 81)	14 (93.3)	23 (82.1)	29 (76.3)	0.39
**Protocol development and assessment**				
Unit protocols mostly based on (n = 88)				
Evidence-based literature	10 (66.7)	18 (58.1)	22 (52.4)	0.74
Both	3 (20.0)	11 (35.5)	15 (35.7)	
Clinicians experience	2 (13.3)	2 (6.4)	5 (11.9)	
Audits to verify adherence to protocols (n = 93)	12 (75.0)	18 (56.3)	20 (44.4)	0.10
Data routinely collected on compliance to protocols (n = 90)	13 (81.3)	18 (60.0)	17 (38.6)	0.01
**Scientific activity**				
Participation in clinical trials (n = 90)	15 (88.2)	19 (61.3)	23 (54.8)	0.048
Meetings to discuss publications (n = 92)	13 (76.5)	14 (43.8)	35 (81.4)	0.002

PPROM: preterm premature rupture of membranes.

Individual characteristics were not associated with unit policy, except for women with in utero transfer who were more often managed in units with a liberal policy (Table 2). Of note, a liberal unit policy was not associated with increased likelihood of receiving a complete course of antenatal steroids.

**Table 2:** Association of individual characteristics and unit policy of tocolysis after PPROM.

Individual characteristics	Unit policy regarding the use of tocolysis after PPROM			p-value
	No-use n = 101 (17 units)	Restricted n = 195 (32 units)	Liberal n = 311 (45 units)	
Mother’s age (years) (n = 604)				
≤20	7 (7.0)	16 (8.2)	13 (4.2)	0.19
21–34	60 (60.0)	129 (66.5)	215 (69.3)	
≥35	33 (33.0)	49 (25.3)	82 (26.5)	
Mother born in Europe (n = 532)	60 (84.5)	127 (74.3)	240 (82.8)	0.054
Nulliparity (n = 605)	46 (45.5)	88 (45.1)	154 (49.8)	0.53
GA at PPROM (weeks) (n = 607)				
24	10 (9.9)	25 (12.8)	35 (11.3)	0.42
25	14 (13.9)	25 (12.8)	57 (18.3)	
26	8 (7.9)	30 (15.4)	50 (16.1)	
27	15 (14.9)	29 (14.9)	43 (13.8)	
28	25 (24.7)	37 (19.0)	52 (16.7)	
29	29 (28.7)	49 (25.1)	74 (23.8)	
In utero transfer (n = 599)	29 (29.6)	74 (38.1)	152 (49.5)	<0.001
Antenatal steroids (n = 594)				
None	1 (1.0)	6 (3.2)	7 (2.3)	0.20
Uncomplete course	4 (4.0)	20 (10.7)	32 (10.4)	
Complete course	95 (95.0)	161 (86.1)	268 (87.3)	
Magnesium sulfate (n = 563)	1 (1.0)	3 (1.7)	7 (2.4)	0.77
Spontaneous onset of labor (n = 602)	60 (59.4)	121 (62.7)	209 (67.9)	0.23
Induction of labor or cesarean before labor because of suspected infection (n = 163)	19 (63.3)	27 (60.0)	40 (45.5)	0.12
Latency duration (days) (n = 607)				
0.5–2	22 (21.8)	48 (24.6)	58 (18.6)	0.45
3–7	38 (37.6)	69 (35.4)	126 (40.5)	
8–14	19 (18.8)	41 (21.0)	72 (23.2)	
15–21	10 (9.9)	17 (8.7)	35 (11.3)	
22–28	8 (7.9)	9 (4.6)	11 (3.5)	
>28	4 (4.0)	11 (5.7)	9 (2.9)	
Mode of delivery (n = 596)				
Vaginal	49 (48.5)	78 (40.9)	136 (44.7)	0.37
Cesarean before labor	32 (31.7)	61 (31.9)	82 (27.0)	
Cesarean during labor	20 (19.8)	52 (27.2)	86 (28.3)	
GA at birth (weeks) (n = 607)				
24	3 (3.0)	11 (5.6)	11 (3.5)	0.31
25	7 (6.9)	11 (5.6)	18 (5.8)	
26	6 (5.9)	19 (9.7)	48 (15.4)	
27	11 (10.9)	21 (10.8)	47 (15.1)	
28	22 (21.8)	38 (19.5)	48 (15.4)	
29	20 (19.8)	45 (23.1)	55 (17.7)	
30	21 (20.8)	35 (18.0)	58 (18.7)	
31	11 (10.9)	15 (7.7)	26 (8.4)	
SGA ≤ 10^th^ (n = 606)*	14 (13.9)	29 (15.0)	62 (19.9)	0.21
Male (n = 606)	61 (60.4)	99 (51.0)	179 (57.6)	0.22

GA: gestational age, PPROM: preterm premature rupture of membranes, SGA: small for gestational age

*According to intrauterine growth curves, based on weight in each country collected in the Euro-Peristat project and modeled using Gardosi’s formula.

### Prolongation of gestation

**Figure 2:**
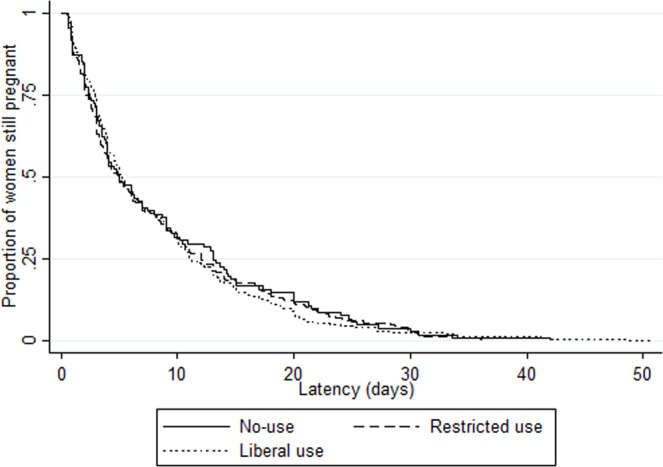
Kaplan Meier survival curves of latency duration by unit policy regarding tocolysis after PPROM. There was no difference in Kaplan-Meier curves of latency duration by unit policy regarding tocolysis after PPROM (logrank test p = 0.88).

**Figure 3:**
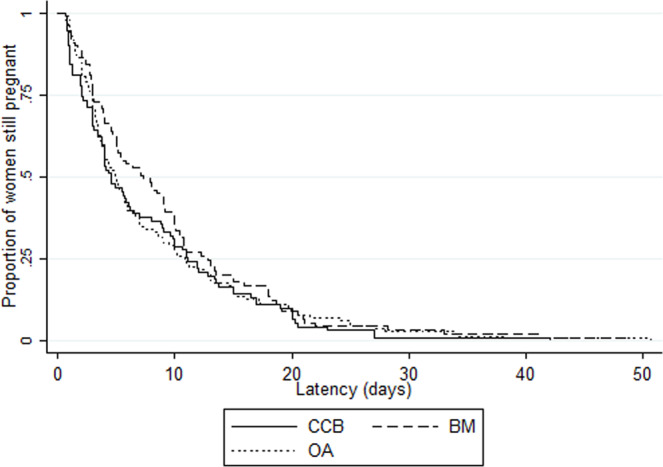
Kaplan Meier survival curves of latency duration by first line tocolytic, in units with a liberal policy of tocolysis after PPROM. BM: betamimetic, CCB: calcium channel blocker, OA: oxytocin antagonist. There was no difference in Kaplan-Meier curves of latency duration by first line tocolytic (logrank test p = 0.43) in units with a liberal policy of tocolysis after PPROM.

**Figure 4:**
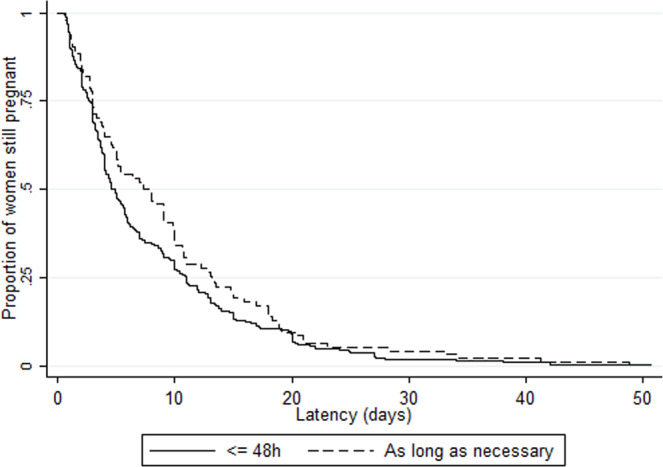
Kaplan Meier survival curves of latency duration by duration of tocolytics use, in units with a liberal policy of tocolysis after PPROM. There was no difference in Kaplan-Meier curves of latency duration by duration of tocolytics use (logrank test p = 0.13) in units with a liberal policy of tocolysis after PPROM.

Overall median latency duration was 5.0 days (IQR 2.6–12.0), with variations by country (from 3.7 [2.0–7.2] in Denmark to 8.5 days [3.0–11.0] in Poland, p = 0.04) as well as by maternity unit (range 0.6 [0.6–0.6] to 21.9 days [9.8–26.0]) (Supplementary Fig. S2). There was no difference in median latency duration by unit policy: 5.0 days (IQR 2.4–13.0), 5.0 (2.1–12.1) and 5.2 (3.0–11.1) with no-use, restricted and liberal policies, respectively (p = 0.87) (Tables 2 and 3). We also found no difference when stratifying by onset of labor: median latency duration by unit policy was 4.0 days (2.4–13.0), 4.3 (2.0–11.1) and 5.0 (2.6–10.4), p = 0.50, in the 390 women with spontaneous labor and 7.0 (3.1–13.6), 5.5 (2.6–13.6), 5.9 (3.1–13.7), p = 0.43, in the 212 women with induction of labor or cesarean before labor. There was no difference in Kaplan-Meier curves of latency duration by unit policy regarding tocolysis after PPROM (logrank test p = 0.88), by first line tocolytic (p = 0.43) or by duration of use (p = 0.13) (Figs. 2–4). Accordingly, multilevel survival analysis did not show any association between unit policies and latency duration after stratifying Cox models for gestational age at PPROM (Table 3).

**Table 3:** Association of unit policy regarding tocolysis after PPROM with latency duration (i.e. time to delivery period).

Unit policy	Latency duration Median (IQR)	Adjusted hazard ratio^†^ (95%CI)
**Unit policy regarding tocolysis after PPROM (n = 607)**		
No-use	5.0 (2.4–13.0)	Ref
Restricted	5.0 (2.1–12.1)	1.15 (0.89–1.48)
Liberal	5.2 (3.0–11.1)	1.14 (0.90–1.44)
Unit policy regarding first line tocolytic (n = 302)*		
Calcium channel blockers	4.6 (2.1–11.1)	Ref
Betamimetics	7.3 (3.1–12.8)	0.85 (0.63–1.15)
Oxytocin antagonists	5.0 (3.0–11.0)	0.92 (0.69–1.23)
Unit policy regarding the duration of tocolysis (n = 311)*		
≤48 hrs	4.9 (2.7–11.0)	Ref
As long as necessary	7.6 (3.0–13.2)	0.83 (0.65–1.07)

IQR: interquartile range, PPROM: preterm premature rupture of membranes

*Among units with liberal policy regarding tocolysis after PPROM

^†^Stratified Cox models (stratification for gestational age at PPROM), with maternity units as level 2 and countries as level 3, and log-normal distribution of the hazard function.

### Perinatal and two-year outcomes

Overall proportions of early-onset sepsis, survival at discharge, survival at discharge without severe morbidity and survival at two years old without gross motor impairment were 7.0%, 90.4%, 77.6% and 87.0%, with variations by country (Supplementary Table S2). There was no case of severe hearing or visual impairment at two years of corrected age in our sample.

After multiple imputation for missing data, the respective proportions of perinatal outcomes with no-use, restricted and liberal policies were as follows: early-onset sepsis (8.1%, 5.9% and 7.4% of infants admitted to NICU), survival at discharge (91.1%, 93.3% and 88.4%), survival at discharge without severe morbidity (77.2%, 80.5% and 75.9%), and survival at two years old without gross motor impairment (88.1%, 91.3% and 83.9%) (Table 4). There was no difference in the frequency of late-onset sepsis among infants admitted to NICU by unit policy (26.3%, 27.0% and 26.4%, respectively, p = 0.99) (Supplementary Table S3). Multilevel logistic regression models adjusted for unit size and gestational age at PPROM did not show any difference in neonatal outcomes by unit policy regarding the use of tocolysis after PPROM (Table 4, Supplementary Tables S3 and S4). Among units with a liberal policy for tocolysis after PPROM, policies regarding the first line tocolytic treatment and the duration of use were not found associated with improved outcomes.

**Table 4:** Association of unit policy of tocolysis after PPROM and perinatal and 2-year outcomes.

Unit policies	Outcome (complete cases) n (%)	Outcome (multiple imputation) (%) [95% CI]	Bivariate analysis* OR (95% CI)	Multivariate analysis† aOR (95% CI)
**Early-onset sepsis among infants admitted to NICU**				
Use of tocolysis	n = 587	n = 594	n = 594	n = 594
No-use	8 (8.2)	(8.1) [2.7–13.5]	Ref	Ref
Restricted	11 (5.8)	(5.9) [2.5–9.2]	0.71 (0.27–1.83)	0.72 (0.25–2.06)
Liberal	22 (7.3)	(7.4) [4.4–10.3]	0.90 (0.39–2.11)	0.78 (0.30–2.04)
First line tocolytic^‡^	n = 291	n = 294	n = 294	n = 294
CCB	3 (3.6)	(3.8) [0.0–8.1]	Ref	Ref
BM	10 (11.6)	(11.6) [4.8–18.5]	3.33 (0.89–12.52)	5.11 (0.84–31.05)
OA	9 (7.4)	(7.4) [2.7–12.1]	2.02 (0.53–7.65)	2.78 (0.59–13.20)
Duration of use^‡^	n = 300	n = 303	n = 303	N = 303
≤48 hrs	14 (6.7)	(6.7) [3.3–10.1]	Ref	Ref
As long as necessary	8 (8.9)	(8.9) [3.0–14.8]	1.36 (0.55–3.36)	1.16 (0.39–3.44)
**Survival at discharge**				
Use of tocolysis	n = 607	n = 607	n = 607	n = 607
No-use	92 (91.1)	(91.1) [85.5–96.7]	Ref	Ref
Restricted	182 (93.3)	(93.3) [89.8–96.9]	1.47 (0.58–3.71)	1.49 (0.49–4.51)
Liberal	275 (88.4)	(88.4) [84.9–92.0]	1.01 (0.43–2.37)	1.31 (0.49–3.53)
First line tocolytic^‡^	n = 302	n = 302	n = 302	n = 302
CCB	80 (88.9)	(88.9) [82.3–95.4]	Ref	Ref
BM	75 (84.3)	(84.3) [76.6–91.9]	0.66 (0.25–1.72)	1.13 (0.38–3.36)
OA	112 (91.1)	(91.1) [86.0–96.1]	1.24 (0.47–3.28)	0.86 (0.29–2.52)
Duration of use^‡^	n = 311	n = 311	n = 311	n = 311
≤48 hrs	196 (90.3)	(90.3) [86.4–94.3]	Ref	Ref
As long as necessary	79 (84.0)	(84.0) [76.6–91.5]	0.57 (0.27–1.21)	0.96 (0.39–2.38)
**Survival at discharge without severe morbidity**				
Use of tocolysis	n = 588	n = 607	n = 607	n = 607
No-use	78 (78.8)	(77.2) [69.0–85.5]	Ref	Ref
Restricted	157 (84.9)	(80.5) [74.9–86.1]	1.21 (0.66–2.21)	1.11 (0.55–2.22)
Liberal	236 (77.6)	(75.9) [71.1–80.7]	0.93 (0.54–1.62)	0.94 (0.49–1.81)
First line tocolytic^‡^	n = 295	n = 302	n = 302	n = 302
CCB	68 (77.3)	(75.6) [66.6–84.5]	Ref	Ref
BM	65 (73.0)	(73.0) [63.7–82.3]	0.90 (0.41–2.00)	1.58 (0.64–3.91)
OA	95 (80.5)	(77.2) [69.8–84.7]	1.14 (0.55–2.37)	1.11 (0.47–2.66)
Duration of use^‡^	n = 304	n = 311	n = 311	n = 311
≤48 hrs	168 (80.0)	(77.4) [71.8–83.0]	Ref	Ref
As long as necessary	68 (72.3)	(72.3) [63.2–81.5]	0.75 (0.39–1.42)	1.17 (0.55–2.48)
**Survival at 2 years corrected age without gross motor impairment**				
Use of tocolysis	n = 400	n = 607	n = 607	n = 607
No-use	46 (79.3)	(88.1) [81.8–94.5]	Ref	Ref
Restricted	94 (84.7)	(91.3) [87.3–95.3]	1.54 (0.68–3.48)	2.13 (0.81–5.59)
Liberal	181 (78.4)	(83.9) [79.8–88.0]	0.90 (0.43–1.91)	1.34 (0.56–3.20)
First line tocolytic^‡^	n = 222	n = 302	n = 302	n = 302
CCB	65 (82.3)	(84.4) [76.9–92.0]	Ref	Ref
BM	48 (69.6)	(76.4) [67.5–85.3]	0.60 (0.28–1.26)	0.79 (0.30–2.05)
OA	60 (81.1)	(88.6) [83.0–94.3]	1.43 (0.65–3.18)	0.86 (0.33–2.20)
Duration of use^‡^	n = 231	n = 311	n = 311	n = 311
≤48 hrs	129 (82.2)	(87.1) [82.6–91.6]	Ref	Ref
As long as necessary	52 (70.3)	(76.6) [68.0–85.2]	0.48 (0.26–0.90)	0.73 (0.33–1.59)

BM: Betamimetics, CCB: Calcium channel blockers, NICU: neonatal intensive care unit, OA: Oxytocin antagonists, OR: odds ratio, aOR: adjusted odds ratio.

*Multilevel random-effect logistic regression with maternity unit as level 2 and country as level 3. Multiple imputation.

^†^Multilevel random-effect logistic regression with maternity unit as level 2 and country as level 3, adjusted for unit characteristics (number of births in 2011) and individual characteristics (gestational age at PPROM). Multiple imputation.

^‡^Among units with a liberal policy for tocolysis after PPROM.

## Discussion

Our findings suggest that, among maternity units with evidence-based policies regarding the use of antenatal steroids and antibiotics for PPROM, there are significant variations in policies regarding the use of tocolysis which are not explained by unit or individual characteristics. Compared to a policy of no-use of tocolysis after PPROM, having a liberal or restricted policy is not associated with prolongation of gestation, or with improved neonatal outcomes. Among units with a liberal policy, policies regarding the duration of use or the type of treatment are not associated with prolongation of gestation, or with neonatal outcomes.

The strengths of our study are its prospective population-based design with a large number of women and infants cared for in maternity units where evidence-based practices are implemented in the setting of PPROM. The variability of declared obstetric policies across and within European countries regarding tocolysis after PPROM provides a unique opportunity to investigate and compare the use and the outcomes of this non evidence-based practice, in a “real-life” setting. Using unit policies to assess practices makes it possible to minimize indication biases which are of concern in observational studies. We consider this approach to be complementary to previous studies based on an individual-level approach^18,19^. Moreover, to the best of our knowledge, we are the first to report outcomes at two years of corrected age in this specific context.

However, this study was limited by the lack of individual data about the tocolytic treatment effectively received by women. Although incomplete application of unit protocols might bias results towards the null, we assume that most women are managed accordingly to the unit policy^34^, as confirmed by the high rate of steroids use in our study population. We were also lacking clinical data such as the presence of uterine contractions or cervical status at PPROM diagnosis to adjust our multivariate models, and we could not assess the association between unit policy and intra-uterine infection for the same reason. However, we do not believe that the proportion of women with contractions or short cervix at admission for PPROM would depend on the unit policy. Outcomes at 2 years of corrected age were collected using a parental report. Even though this is not the gold standard, such evaluations based on standard definitions can be a valid, reliable, efficient and cost-effective way to assess neurodevelopmental impairment in preterm infants^24,26,35^. Attrition at 2 years of corrected age was substantial. Although appropriate statistical methods allowed for accounting for missing data related to loss to follow-up and obtaining non biased estimators, these results should be interpreted cautiously. The inclusion of only large units with 10 or more very preterm admissions during the study period in the unit survey led to an underrepresentation of small maternity units. However, only 25 women gave birth in these 13 small units and one can assume that these units often participate in a perinatal network and share common protocols with larger units. Finally, women with PPROM who delivered after 31^6/7^ weeks were not included in the EPICE cohort because of the study’s design. To mitigate this right-truncation bias, we studied only cases of PPROM at 24^0/7^–29^6/7^ weeks, but likely missed some cases with the longest latency durations (known to be related to low gestational age at PPROM, but not to tocolysis)^18,36^, and the best prognosis.

Wide variations in unit policies and practices regarding tocolysis after PPROM have been previously reported in the United States^15,16^, Australia^37^, France^17,38^ and Canada^39^, with no-use ranging from 6 to 27%, use restricted to women with contractions ranging from 56 to 62%, liberal use ranging from 31 to 75%, and 48h-use ranging from 72 to 94%. These variations can reflect the evolution of practices over time, and more likely the lack of consensus in international guidelines driven by insufficient evidence in the scientific literature^40^.

Our findings regarding neonatal outcomes are in line with recent publications. A meta-analysis based on 8 randomized controlled trials (n = 408 women with PPROM) showed that, as compared to no tocolysis, tocolysis after PPROM was not associated with a significant effect on perinatal mortality, neonatal sepsis, necrotizing enterocolitis or intraventricular hemorrhage although significantly associated with prolongation of gestation^19^. The authors emphasized that the effect of tocolysis in women who receive both antibiotics and steroids remained unanswered, due to the design of available studies (most did not administer evidence-based treatments), small sample sizes and limited quality. An observational study, based on a recent prospective population-based cohort of preterm infants, and including 803 women with PPROM (of whom 73%, 89% and 96% received tocolysis, steroids and antibiotics, respectively), showed that tocolysis was not associated with survival without severe morbidity or latency ≥48 hours^18^. This study based on individual data and the present findings based on unit policies are complementary and, although their observational design does not allow a causal interpretation, they support the hypothesis that tocolysis might not provide further benefits when evidence-based interventions are implemented.

Finally, it should be noted that tocolytics can have side-effects and possibly long term consequences, although this has been barely assessed, either directly as they cross the placenta to the fetus or by prolonging fetal exposure to inflammation^41,42^. If balancing the benefits and harms of treatments administered to women at risk of preterm birth remains a challenge in daily practice, minimizing non evidence-based interventions or policies that provide little or no benefit to patients seems reasonable.

In this light, a randomized controlled trial adequately powered to assess the impact of tocolysis on neonatal and 2-year outcomes in the scope of current obstetric practices would be needed to establish best practice.

## Conclusion

Compared to a unit policy of no-use of tocolysis after PPROM, having a liberal or restricted policy is not associated with prolongation of gestation, nor with improved neonatal outcomes. These results should be confirmed by new studies based on individual data, with sufficient details on tocolytic treatment, clinical characteristics and other interventions such as magnesium sulfate for neuroprotection. However, this study adds to the increasing body of evidence on the absence of obstetric or neonatal benefits associated with the use of tocolysis after PPROM, specifically when evidence-based treatments are routinely offered.

## Supplementary information

Supplementary figures and tables.

## Data Availability

The datasets generated during and/or analyzed during the current study are available from the PI of the cohort study (Jennifer Zeitlin) on reasonable request.
