# Supplementary material for: Unit policies regarding tocolysis after preterm premature rupture of membranes: association with latency, neonatal and 2-year outcomes (EPICE cohort)
Source: Sci Rep. 2020 Jun 12;10:9535. doi: 10.1038/s41598-020-65201-y (PMC7293322; doi:10.1038/s41598-020-65201-y)
Supplement: Supplementary file 1 — Supplementary figures and tables. [file 41598_2020_65201_MOESM1_ESM.docx]

**Unit policies regarding tocolysis after preterm premature rupture of membranes: association with latency, neonatal and 2-year outcomes (EPICE cohort)**

Elsa LORTHE^1*^, RM, PhD, Carla MOREIRA^2^, PhD, Tom WEBER^3^, MD, DMedSci, Lene D. HUUSOM^4^, MD, Stephan SCHMIDT^5^, MD, PhD, Rolf F. MAIER^6^, MD, Pierre-Henri JARREAU^7,8^, MD, PhD, Marina CUTTINI^9^, MD, PhD, Elizabeth S. DRAPER^10^, MPhil PhD, Jennifer ZEITLIN^7^, PhD, Henrique BARROS^1,11^, MD, PhD, on behalf of the EPICE research group

**Supplementary figures and tables**

**
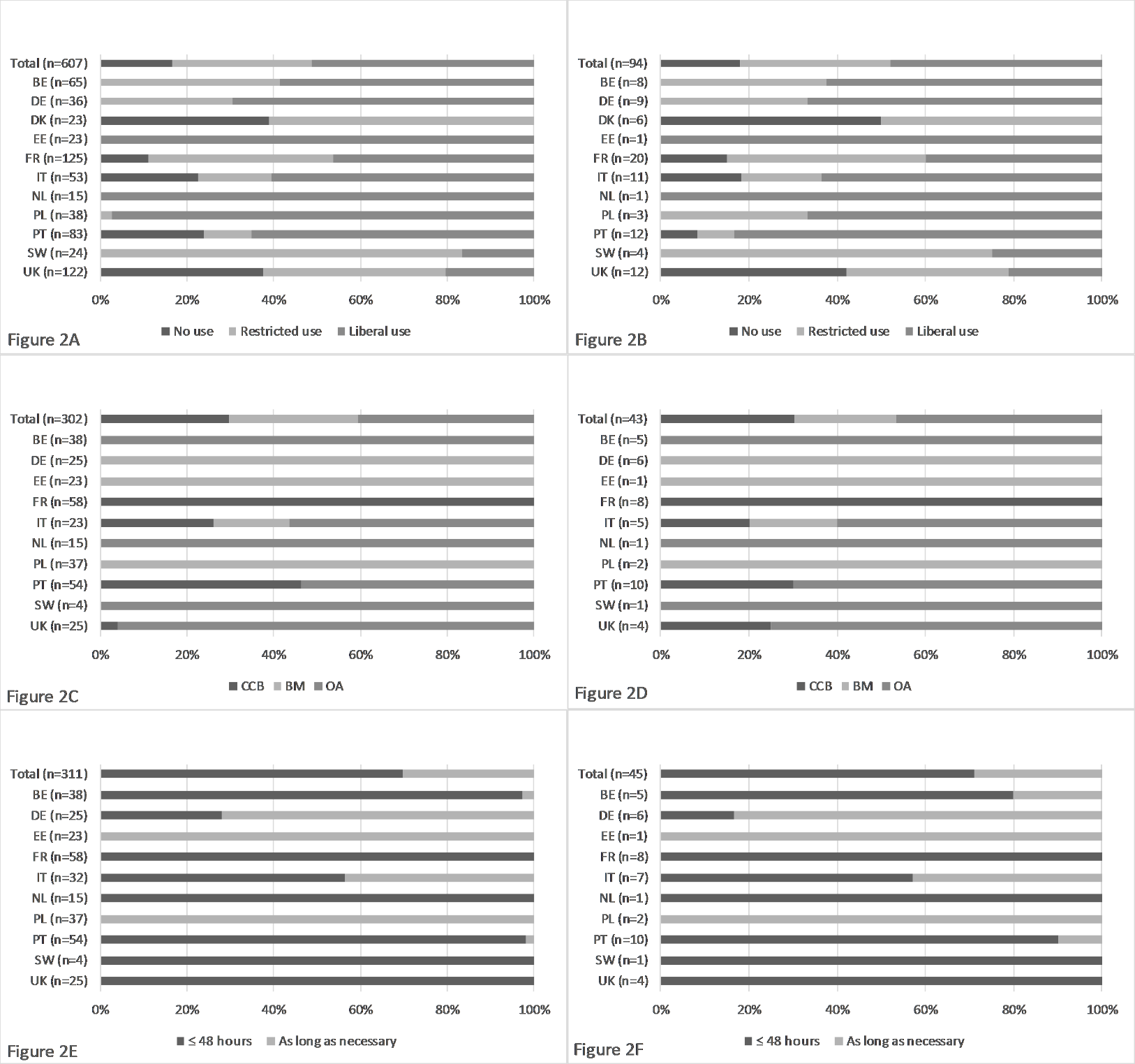
**

**Figure S1: Unit policies by country**

This figure displays unit policies by country, regarding

[2A] the use of tocolysis after PPROM (607 individuals)

[2B] the use of tocolysis after PPROM (94 units)

[2C] First line tocolytic treatment in units with a liberal policy (302 individuals)

[2D] First line tocolytic treatment in units with a liberal policy (43 units)

[2E] Duration of tocolysis use in units with a liberal policy (311 individuals)

[2F] Duration of tocolysis use in units with a liberal policy (45 units)


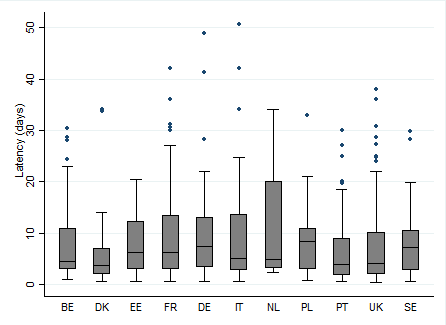


**Figure S2: Box plot of latency duration by country**

BE: Belgium (n=65), DK: Denmark (n=23), EE: Estonia (n=23), FR: France (n=125), DE: Germany (n=36), IT: Italy (n=53), NL: Netherlands (n=15), PL: Poland (n=38), PT: Portugal (n=83), UK: United Kingdom (n=122), SE: Sweden (n=24)

Supplementary Table S1: Comparison of unit and individual characteristics without and with follow-up at 2 years corrected age.

|  | | | Follow-up at 2 years among infants alive at 2 years old | |  |
| --- | --- | --- | --- | --- | --- |
|  | | | Without (n=198) | With (n=347) | P-value* |
| **Country** (n=545) | | |  |  | <.001 |
|  | Belgium | | 34 (17.2) | 26 (7.5) |  |
|  | Denmark | | 5 (2.5) | 17 (4.9) |  |
|  | Estonia | | 0 (0) | 21 (6.0) |  |
|  | France | | 15 (7.6) | 95 (27.4) |  |
|  | Germany | | 17 (8.6) | 14 (4.0) |  |
|  | Italy | | 10 (5.1) | 36 (10.4) |  |
|  | Netherlands | | 5 (2.5) | 10 (2.9) |  |
|  | Poland | | 7 (3.5) | 20 (5.8) |  |
|  | Portugal | | 23 (11.6) | 49 (14.1) |  |
|  | United Kingdom | | 74 (37.4) | 44 (12.7) |  |
|  | Sweden | | 8 (4.0) | 15 (4.3) |  |
| **Unit characteristics** | | |  |  |  |
| Level III maternity unit (n=545) | | | 177 (89.4) | 313 (90.2) | .19 |
| Public status (n=543) | | | 176 (89.8) | 329 (94.8) | .65 |
| Unit policy (n=545) | | |  |  |  |
|  | | No-use | 42 (21.2) | 50 (14.4) | .01 |
|  | | Restricted | 81 (40.9) | 101 (29.1) |  |
|  | | Liberal | 75 (37.9) | 196 (56.5) |  |
| First line tocolytic† (n=263) | | |  |  |  |
|  | | CCB | 10 (13.3) | 69 (36.7) | .44 |
|  | | BM | 19 (25.3) | 54 (28.7) |  |
|  | | OA | 46 (61.3) | 65 (34.6) |  |
| Duration of use† (n=271) | | |  |  |  |
|  | | ≤ 48 hrs | 56 (74.7) | 137 (69.9) | .66 |
|  | | As long as necessary | 19 (25.3) | 59 (30.1) |  |
| **Individual characteristics** | | |  |  |  |
| Mother’s age (years) (n=542) | | |  |  |  |
|  | | ≤20 | 23 (11.7) | 10 (2.9) | <.001 |
|  | | 21-34 | 139 (70.9) | 230 (66.5) |  |
|  | | ≥35 | 34 (17.4) | 106 (30.6) |  |
| Mother born in Europe (n=476) | | | 99 (76.7) | 283 (81.6) | .04 |
| Nulliparity (n=543) | | | 75 (37.9) | 189 (54.8) | <.001 |
| In utero transfer (n=540) | | | 71 (36.4) | 159 (46.1) | .65 |
| Antenatal steroids (n=534) | | |  |  |  |
|  | | None | 4 (2.1) | 7 (2.0) | .47 |
|  | | Uncomplete course | 12 (6.2) | 35 (10.3) |  |
|  | | Complete course | 177 (91.7) | 299 (87.7) |  |
| Spontaneous onset of labor (n=540) | | | 138 (70.1) | 214 (62.4) | .54 |
| Mode of delivery (n=534) | | |  |  |  |
|  | | Vaginal | 81 (41.8) | 154 (45.3) | .58 |
|  | | Cesarean before labor | 53 (27.3) | 105 (30.9) |  |
|  | | Cesarean during labor | 60 (30.9) | 81 (23.8) |  |
| Male (n=545) | | | 108 (54.6) | 194 (55.9) | .45 |
| SGA≤10^th^ (n=545) | | | 41 (20.7) | 49 (14.1) | .07 |
| GA at PPROM (weeks) (n=545) | | |  |  |  |
|  | | 24 | 16 (8.1) | 37 (10.7) | .78 |
|  | | 25 | 27 (13.6) | 47 (13.5) |  |
|  | | 26 | 30 (15.2) | 48 (13.8) |  |
|  | | 27 | 28 (14.1) | 54 (15.6) |  |
|  | | 28 | 43 (21.7) | 67 (19.3) |  |
|  | | 29 | 54 (27.3) | 94 (27.1) |  |
| Latency duration (days) (n=545) | | |  |  |  |
|  | | 0.5-2 | 39 (19.7) | 69 (19.9) | .62 |
|  | | 3-7 | 79 (39.9) | 134 (38.6) |  |
|  | | 8-14 | 47 (23.7) | 74 (21.3) |  |
|  | | 15-21 | 18 (9.1) | 38 (11.0) |  |
|  | | 22-28 | 9 (4.6) | 16 (4.6) |  |
|  | | >28 | 6 (3.0) | 16 (4.6) |  |
| GA at birth (weeks) (n=545) | | |  |  |  |
|  | | 24 | 9 (4.6) | 11 (3.2) | .86 |
|  | | 25 | 7 (3.5) | 14 (4.0) |  |
|  | | 26 | 23 (11.6) | 38 (11.0) |  |
|  | | 27 | 19 (9.6) | 48 (13.8) |  |
|  | | 28 | 34 (17.2) | 63 (18.2) |  |
|  | | 29 | 47 (23.7) | 68 (19.6) |  |
|  | | 30 | 41 (20.7) | 73 (21.0) |  |
|  | | 31 | 18 (9.1) | 32 (9.2) |  |
| **Outcomes** | | |  |  |  |
| Early-onset sepsis (n=539) | | | 8 (4.1) | 23 (6.7) | .06 |
| Severe morbidity at discharge (n=526) | | | 21 (11.3) | 37 (10.9) | .68 |

BM: Betamimetics, CCB: Calcium channel blockers, GA: gestational age, OA: Oxytocin antagonists, PPROM: preterm premature rupture of membranes, SGA: small for gestational age

* P-value obtained after taking into account unit and country (multilevel models)

† Among units with a liberal policy

Supplementary Table S2: Outcomes by country

| Country | Early-onset sepsis* | Survival at discharge | Survival at discharge without severe morbidity | Survival at two-years without gross motor impairment |
| --- | --- | --- | --- | --- |
| (Complete cases) | n (%)  (n=587) | n (%) (n=607) | n (%)  (n=588) | n (%)  (n=400) |
| Total (n=607) | 41 (7.0) | 549 (90.4) | 471 (80.1) | 321 (80.3) |
| Belgium (n=65) | 7 (11.1) | 60 (92.3) | 52 (86.7) | 25 (83.3) |
| Denmark (n=23) | 1 (4.8) | 22 (95.7) | 19 (86.4) | 17 (94.4) |
| Estonia (n=23) | 3 (13.0) | 22 (95.7) | 20 (87.0) | 18 (78.3) |
| France (n=125) | 4 (3.5) | 111 (88.8) | 100 (82.0) | 86 (79.6) |
| Germany (n=36) | 3 (8.8) | 32 (88.9) | 27 (79.4) | 14 (73.7) |
| Italy (n=53) | 4 (7.7) | 47 (88.7) | 44 (83.0) | 35 (81.4) |
| Netherlands (n=15) | 1 (6.7) | 15 (100) | 13 (86.7) | 10 (100) |
| Poland (n=38) | 4 (11.1) | 27 (71.1) | 22 (57.9) | 17 (56.7) |
| Portugal (n=83) | 3 (3.6) | 72 (86.8) | 61 (73.5) | 48 (81.4) |
| UK (n=122) | 10 (8.3) | 118 (96.7) | 92 (80.0) | 37 (82.2) |
| Sweden (n=24) | 1 (4.2) | 23 (95.8) | 21 (91.3) | 14 (93.3) |
| p-value | p=0.58 | p=0.002 | p=0.03 | p=0.06 |
| (Multiple imputation) | % (95%CI) (n=600) | % (95%CI) (n=607) | % (95%CI) (n=607) | % (95%CI)  (n=607) |
| Total (n=607) | 7.0 (4.9-9.1) | 90.4 (88.1-92.8) | 77.6 (74.3-80.9) | 87.0 (84.3-89.7) |
| Belgium (n=65) | 11.1 (3.1-19.1) | 92.3 (85.6-99.0) | 80.0 (70.0-90.0) | 92.3 (85.6-99.0) |
| Denmark (n=23) | 4.5 (0.0-13.9) | 95.7 (86.6-100.0) | 82.6 (65.8-99.5) | 95.7 (86.6-96.6) |
| Estonia (n=23) | 13.0 (0.0-28.0) | 95.7 (86.6-100.0) | 87.0 (72.0-100.0) | 78.3 (59.9-96.6) |
| France (n=125) | 3.6 (0.1-7.2) | 88.8 (83.2-94.4) | 80.0 (72.9-87.1) | 82.4 (75.6-89.2) |
| Germany (n=36) | 9.1 (0.0-19.4) | 88.9 (78.1-99.7) | 75.0 (60.1-89.9) | 86.1 (74.2-98.0) |
| Italy (n=53) | 7.7 (0.2-15.2) | 88.7 (80.0-97.5) | 83.0 (72.6-93.5) | 84.9 (74.9-94.9) |
| Netherlands (n=15) | 6.7 (0.0-21.1) | 100 | 86.7 (66.9-100.0) | 100 |
| Poland (n=38) | 11.1 (0.3-21.9) | 71.1 (55.9-86.2) | 57.9 (41.4-74.4) | 65.8 (50.0-81.6) |
| Portugal (n=83) | 3.6 (0.0-7.7) | 86.7 (79.3-94.2) | 73.5 (63.8-83.2) | 86.7 (79.3-94.2) |
| UK (n=122) | 8.3 (3.3-13.2) | 96.7 (93.5-99.9) | 75.4 (67.7-83.2) | 93.4 (89.0-97.9) |
| Sweden (n=24) | 4.2 (0.0-12.8) | 95.8 (87.2-100.0) | 87.5 (73.2-100.0) | 95.8 (87.2-100.0) |

NICU: neonatal intensive care unit, UK: United Kingdom

* Among infants admitted to NICU

Supplementary Table S3: Perinatal and 2-years outcomes by unit policy regarding tocolysis after PPROM (complete cases analyses)

|  | | Unit policy regarding the use of tocolysis after PPROM | | |  |
| --- | --- | --- | --- | --- | --- |
| Outcome | | No-use  n=101 (17 units) | Restricted  n=195 (32 units) | Liberal  n=311 (45 units) | p-value |
| **Vital status at birth** (n=607) | |  |  |  |  |
|  | Stillbirth | 1 (1.0) | 3 (1.5) | 5 (1.6) | .99 |
|  | Labor ward death | 0 (0.0) | 1 (0.5) | 3 (1.0) |  |
|  | Live birth | 100 (99.0) | 191 (98.0) | 303 (97.4) |  |
| **Early-onset sepsis among infants admitted to NICU** (n=587) | | 8 (8.2) | 11 (5.8) | 22 (7.3) | .72 |
| **Late-onset sepsis among infants admitted to NICU** (n=583) | | 26 (26.3) | 50 (27.0) | 79 (26.4) | .99 |
| **Survival at discharge** (n=607) | | 92 (91.1) | 182 (93.3) | 275 (88.4) | .18 |
| **Survival at discharge without:** | |  |  |  |  |
|  | EOS (n=601) | 85 (85.0) | 169 (87.6) | 258 (83.8) | .51 |
|  | LOS (n=596) | 69 (69.0) | 127 (67.2) | 199 (64.8) | .71 |
|  | Severe IVH (n=600) | 83 (83.0) | 171 (89.5) | 262 (84.8) | .21 |
|  | Cystic PVL (n=600) | 86 (86.0) | 173 (91.1) | 265 (85.5) | .17 |
|  | Severe NEC (n=607) | 92 (91.1) | 182 (93.3) | 270 (86.8) | .06 |
|  | Severe ROP (n=596) | 90 (90.0) | 172 (91.0) | 256 (83.4) | .03 |
|  | Severe BPD (n=493) | 43 (81.1) | 148 (90.8) | 237 (85.6) | .13 |
|  | Any severe morbidity* (n=589) | 78 (78.8) | 157 (84.9) | 236 (77.6) | .14 |
| **Outcomes at 2 years corrected age** | | |  |  |  |
| Death after discharge among infants discharged alive (n=549) | | 0 (0) | 0 (0) | 4 (1.5) | .13 |
| Follow-up at 2 years among infants alive at 2 years (n=545) | | 50 (54.4) | 101 (55.5) | 196 (72.3) | <.001 |
| Gross motor impairment (n=343) | | 3 (6.1) | 4 (4.0) | 10 (5.2) | .78 |
| Hearing impairment (n=343) | | 0 | 0 | 0 | - |
| Visual impairment (n=342) | | 0 | 0 | 0 | - |
| Survival at 2 years corrected age without gross motor impairment among all eligible fetuses (n=400) | | 46 (79.3) | 94 (84.7) | 181 (78.4) | .38 |

BPD: bronchopulmonary dysplasia, EOS: early-onset sepsis, IVH: intra-ventricular hemorrhage, LOS: late-onset sepsis, NEC: necrotizing enterocolitis, NICU: neonatal intensive care unit, PPROM: preterm premature rupture of membranes, PVL: periventricular leukomalacia, ROP: retinopathy of prematurity

* Any severe morbidity defined as survival at discharge without severe IVH, cystic PVL, severe NEC or severe ROP.

Supplementary Table S4: Association of unit’s policy of tocolysis after PPROM and perinatal and 2-years outcomes (complete cases analyses)

| Unit policies | | Outcome  n (%) | Bivariate analysis*  OR (95% CI) | Multivariate analysis†  aOR (95% CI) |
| --- | --- | --- | --- | --- |
|  | | **Early-onset sepsis among infants admitted to NICU** | | |
| Use of tocolysis | | n=587 | n=587, ICC_2_=10^-5^ / ICC_3_=10^-5^ | n=587, ICC_2_=10^-34^/ ICC_3_=10^-33^ |
|  | No-use | 8 (8.2) | Ref | Ref |
|  | Restricted | 11 (5.8) | 0.70 (0.27-1.79) | 0.71 (0.25-2.06) |
|  | Liberal | 22 (7.3) | 0.89 (0.38-2.07) | 0.77 (0.29-2.02) |
| First line tocolytic^‡^ | | n=291 | n=291, ICC_2_=10^-33^/ ICC_3_=10^-34^ | n=291, ICC_2_=10^-33^/ ICC_3_=10^-36^ |
|  | CCB | 3 (3.6) | Ref | Ref |
|  | BM | 10 (11.6) | 3.51 (0.93-13.24) | 6.02 (1.04-34.99) |
|  | OA | 9 (7.4) | 2.12 (0.56-8.09) | 3.09 (0.66-14.50) |
| Duration of use^‡^ | | n=300 | n=300, ICC_2_=10^-30^/ ICC_3_=10^-32^ | n=300, ICC_2_=10^-33^/ ICC_3_=10^-34^ |
|  | ≤ 48 hrs | 14 (6.7) | Ref | Ref |
|  | As long as necessary | 8 (8.9) | 1.37 (0.55-3.38) | 1.22 (0.42-3.57) |
|  | | **Survival at discharge** | |  |
| Use of tocolysis | | n=607 | n=607, ICC_2_=0.08/ ICC_3_=0.08 | n=607, ICC_2_=0.12/ ICC_3_=0.12 |
|  | No-use | 92 (91.1) | Ref | Ref |
|  | Restricted | 182 (93.3) | 1.47 (0.58-3.71) | 1.49 (0.49-4.51) |
|  | Liberal | 275 (88.4) | 1.01 (0.43-2.37) | 1.31 (0.49-3.53) |
| First line tocolytic^‡^ | | n=302 | n=302, ICC_2_=0.01 /ICC_3_=0.01 | n=302, ICC_2_=10^-33^/ICC_3_=10^-33^ |
|  | CCB | 80 (88.9) | Ref | Ref |
|  | BM | 75 (84.3) | 0.66 (0.25-1.72) | 1.13 (0.38-3.36) |
|  | OA | 112 (91.1) | 1.24 (0.47-3.28) | 0.86 (0.29-2.52) |
| Duration of use^‡^ | | n=311 | n=311, ICC_2_=0.01 /ICC_3_=0.01 | n=311, ICC_2_=10^-34^/ ICC_3_=10^-35^ |
|  | ≤ 48 hrs | 196 (90.3) | Ref | Ref |
|  | As long as necessary | 79 (84.0) | 0.57 (0.27-1.21) | 0.96 (0.39-2.38) |
|  | | **Survival at discharge without severe morbidity** | | |
| Use of tocolysis | | n=588 | n=588, ICC_2_=0.01/ICC_3_=0.01 | n=588, ICC_2_=0.04 / ICC_3_=0.04 |
|  | No-use | 78 (78.8) | Ref | Ref |
|  | Restricted | 157 (84.9) | 1.45 (0.76-2.80) | 1.30 (0.60-2.81) |
|  | Liberal | 236 (77.6) | 0.93 (0.53-1.65) | 0.96 (0.48-1.92) |
| First line tocolytic^‡^ | | n=295 | n=295, ICC_2_=0.02/ICC_3_=0.02 | n=295, ICC_2_=0.02 / ICC_3_=0.02 |
|  | CCB | 68 (77.3) | Ref | Ref |
|  | BM | 65 (73.0) | 0.81 (0.37-1.78) | 1.36 (0.48-3.83) |
|  | OA | 95 (80.5) | 1.24 (0.59-2.59) | 1.23 (0.48-3.15) |
| Duration of use^‡^ | | n=304 | n=304, ICC_2_=0.02/ICC_3_=0.02 | n=304, ICC_2_=0.02 / ICC_3_=0.02 |
|  | ≤ 48 hrs | 168 (80.0) | Ref | Ref |
|  | As long as necessary | 68 (72.3) | 0.65 (0.34-1.22) | 0.92 (0.41-2.06) |
|  | | **Survival at 2 years corrected age without gross motor impairment** | | |
| Use of tocolysis | | n=400 | n=400, ICC_2_=0.03/ICC_3_=10^-33^ | n=400, ICC_2_=10^-35^ / ICC_3_=10^-35^ |
|  | No-use | 46 (79.3) | Ref | Ref |
|  | Restricted | 94 (84.7) | 1.40 (0.59-3.31) | 1.53 (0.60-3.91) |
|  | Liberal | 181 (78.4) | 0.93 (0.44-1.97) | 1.02 (0.46-2.27) |
| First line tocolytic^‡^ | | n=222 | n=222, ICC_2_=10^-32^/ICC_3_=10^-33^ | n=222, ICC_2_=10^-39^ / ICC_3_=10^-54^ |
|  | CCB | 65 (82.3) | Ref | Ref |
|  | BM | 48 (69.6) | 0.49 (0.23-1.07) | 0.78 (0.29-2.09) |
|  | OA | 60 (81.1) | 0.92 (0.41-2.10) | 0.45 (0.17-1.23) |
| Duration of use^‡^ | | n=231 | n=231, ICC_2_=10^-34^/ICC_3_=10^-34^ | n=231, ICC_2_=10^-33^ / ICC_3_=10^-35^ |
|  | ≤ 48 hrs | 129 (82.2) | Ref | Ref |
|  | As long as necessary | 52 (70.3) | 0.51 (0.27-0.98) | 1.02 (0.43-2.39) |

BM: Betamimetics, CCB: Calcium channel blockers, ICC: intra class correlation, NICU: neonatal intensive care unit, OA: Oxytocin antagonists, OR: odds ratio, aOR: adjusted odds ratio

* Multilevel random-effect logistic regression with maternity unit as level 2 (ICC_2_) and country as level 3 (ICC_3_). Complete-cases analysis.

† Multilevel random-effect logistic regression with maternity unit as level 2 (ICC_2_) and country as level 3 (ICC_3_), adjusted for unit characteristics (number of births in 2011) and individual characteristics (gestational age at PPROM). Complete-cases analysis.

‡ Among units with a liberal policy for tocolysis after PPROM.
